# The intracellular trafficking mechanism of Lipofectamine-based transfection reagents and its implication for gene delivery

**DOI:** 10.1038/srep25879

**Published:** 2016-05-11

**Authors:** Francesco Cardarelli, Luca Digiacomo, Cristina Marchini, Augusto Amici, Fabrizio Salomone, Giuseppe Fiume, Alessandro Rossetta, Enrico Gratton, Daniela Pozzi, Giulio Caracciolo

**Affiliations:** 1 Center for Nanotechnology Innovation @NEST, Istituto Italiano di Tecnologia, Piazza San Silvestro 12, 56127 Pisa, Italy; 2 School of Bioscience and Veterinary Medicine, University of Camerino, Via Gentile III da Varano, 62032 Camerino (MC), Italy; 3 Department of Molecular Medicine, “Sapienza” University of Rome, Viale Regina Elena 291, 00161, Rome, Italy; 4 Laboratory for Fluorescence Dynamics, Department of Biomedical Engineering, University of California, Irvine, 3120 Natural Sciences 2, Irvine, CA 92697-2715, USA

## Abstract

Lipofectamine reagents are widely accepted as “gold-standard” for the safe delivery of exogenous DNA or RNA into cells. Despite this, a satisfactory mechanism-based explanation of their superior efficacy has remained mostly elusive thus far. Here we apply a straightforward combination of live cell imaging, single-particle tracking microscopy, and quantitative transfection-efficiency assays on live cells to unveil the intracellular trafficking mechanism of Lipofectamine/DNA complexes. We find that Lipofectamine, contrary to alternative formulations, is able to efficiently avoid active intracellular transport along microtubules, and the subsequent entrapment and degradation of the payload within acidic/digestive lysosomal compartments. This result is achieved by random Brownian motion of Lipofectamine-containing vesicles within the cytoplasm. We demonstrate here that Brownian diffusion is an efficient route for Lipofectamine/DNA complexes to avoid metabolic degradation, thus leading to optimal transfection. By contrast, active transport along microtubules results in DNA degradation and subsequent poor transfection. Intracellular trafficking, endosomal escape and lysosomal degradation appear therefore as highly interdependent phenomena, in such a way that they should be viewed as a single barrier on the route for efficient transfection. As a matter of fact, they should be evaluated in their entirety for the development of optimized non-viral gene delivery vectors.

It is widely accepted that the final transfection efficiency (TE) of lipid-based transfection reagents (TRs) is rate-limited by several biological barriers such as cellular uptake, intracellular trafficking, endosomal escape and nuclear entry^1^,^2^,^3^,^4^,^5^,^6^,^7^,^8^. In this context, since their launch in 1993, Lipofectamine (LFN) reagents have become the most used TRs, with over 50,000 references to date. Due to their high TE for both DNA and siRNA/miRNA across a broad range of cell lines, including difficult-to-transfect cells, they are considered the “gold-standard” among TRs and are routinely used as a basis of comparison to evaluate the efficiencies of alternative transfection methods. Despite this, however, a clear-cut mechanism-based explanation of LFN superior performances is still missing. In this regard, available data support a role of microtubules in the active, vesicle-mediated, transport of LFN/DNA complexes towards the nucleus, analogously to what happens for some viruses^7^. This is somewhat surprising, however, in light of the clear correlation between active transport along microtubules and final localization of the DNA payload within acidic/digestive lysosomal compartments, as demonstrated for a number of alternative lipid-based formulations^9^,^10^. To tackle this issue here we use a unique combination of live cell imaging, single-particle tracking microscopy, and quantitative transfection-efficiency assays. In particular, an effort was focused in the visualization of the LFN/DNA complexes trafficking in live cells by fluorescence-based single-particle tracking, with no a priori assumption about the mechanism of transport of LFN. To this aim, we used complexes of LFN ‘2000’ with Cy3-labeled plasmidic DNA. On the other hand, lipoplexes made of 1,2-dioleoyl-3-trimethylammonium-propane (DOTAP), dioleoylphosphocholine (DOPC) and Cy3-DNA were selected as a typical low-TE control^11^,^12^. Notably, we found that Lipofectamine, contrary to DOTAP-DOPC, is able to efficiently avoid active intracellular transport along cytoskeleton components, and the subsequent trapping and possible metabvolic degradation of the payload within acidic/digestive lysosomal compartments. This result is made possible by the emergence of a peculiar random Brownian motion of Lipofectamine-containing vesicles within the cytoplasm. We are tempted to argue that this behavior represents a hallmark of the superior transfection efficacy of this formulation. Reported results shall have a profound impact on the guidance for the development of new generations of further optimized non-viral, lipid-based, gene delivery materials.

## Results and Discussion

### Single-particle tracking applied to vesicles: probing the mechanism of transport of LFN in live cells

**Figure 1:**
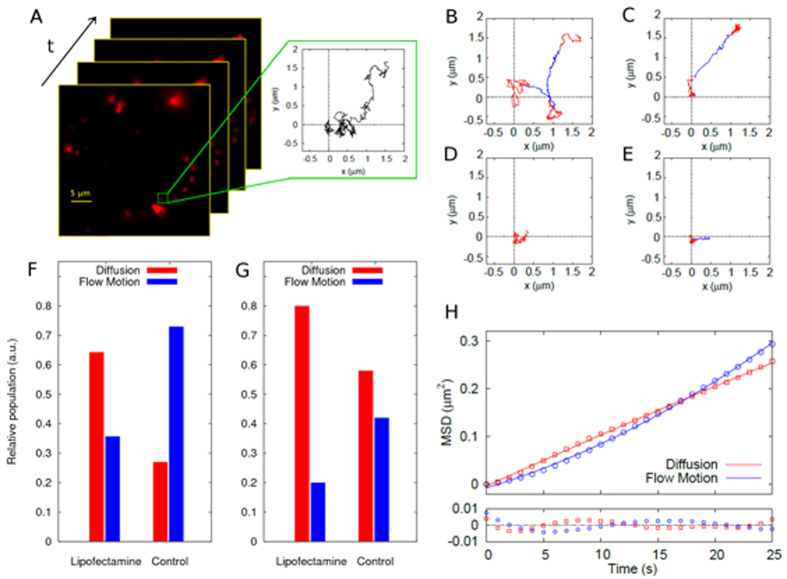
(**A**) Schematic evaluation of a single particle track from a set of confocal images acquired within 300 s, with a time lapse Δt = 1 s. Representative trajectories of complexes in not treated chinese hamster ovarian (CHO) cells: (**B**) Lipofectamine/DNA; (**C**) DOTAP/DOPC/DNA (DD/DNA). Representative trajectories of complexes in nocodazole (NCZ)–treated CHO cells: (**D**) Lipofectamine/DNA and (**E**) DD/DNA. Diffusion (red) and flow motion (blue) segments are shown. Relative populations of the acquired tracks for Lipofectamine and DD in not treated (**F**) and NCZ-treated (**G**) CHO cells. (**H**) Mean square displacement (MSD) analysis of two representative tracks. MSD calculation was used for the measurement of the dynamic parameters, i.e. diffusion coefficients and flow speed.

**Figure 2:**
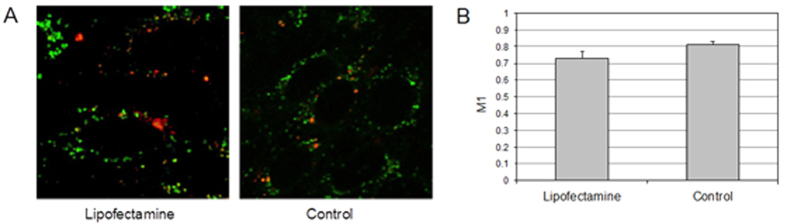
Colocalization of DNA-loaded complexes and endosomes. (**A**) Representative confocal images of Cy3-labeled Lipofectamine/DNA (Lipofectamine) and DOTAP/DOPC/DNA (Control) complexes (‘red’ signal). In both images intracellular vesicles were labeled with the endosomial marker FM4–64 (‘green’ signal). In detail, after 1 h of incubation of cells with lipoplexes, the FM4–63 marker was added following manufacturer’s instructions. After 1 additional hour of incubation, cells were viewed at the confocal microscope. A single laser line (488 nm) was used to excite both fluorophores, whose emission was then recorded in two spectrally-separate channels (550–600 for Cy3 and 650–700 for FM4–64). (**B**) ImageJ software (NIH Image; http://rsbweb.nih.gov/ij/) was used to measure colocalization through Mander’s overlap coefficient (M1 = colocalized red/total red). Manders overlap coefficient M1 was calculated according to Costes *et al*.^36^ As evident, DNA was largely found inside endosomes. No significant differences between Lipofectamine and Control were found. Colocalization measurements were evaluated for not less than 50 complexes for each formulation.

Chinese hamster ovarian (CHO) cells were seeded in 6-wells plates and incubated with LFN/DNA (further experimental details can be found in the Supporting Information). Thirty minutes after complex administration, cells were washed to remove complexes bound to the plasma membrane and the lipoplex trafficking process was monitored in real time for the following four hours. Lipoplexes exhibit distinct mobility phases. They have been deeply investigated by the Braüchle group using single particle tracking in living cells^13^. First lipoplexes are bound to the cell surface and show slow transport behavior (phase I). Once inside the cytosol, lipoplex association with actin results in anomalous and confined diffusion (phase II). Phase III is characterized by active transport along microtubules leading to perinuclear accumulation. In previous studies^14^ some of us demonstrated that, after initial association with actin filaments, lipoplexes need a few hours to reach the perinuclear region (typically 0–4 hours depending on lipid formulation). According to the results of the Braüchle group and to our previous results, we therefore focused on the intracellular motion of lipoplexes (phase III, from thirty minutes to 3 hours after cell administration). Transmission light microscopy shows the presence of a cell in the observation field. As shown in Fig. 1A, LFN/DNA complexes are visible as bright, dot-like fluorescent structures. Regular spot-like structures as those displayed in Fig. 1A are due to intact DNA-loaded complexes, while diffuse fluorescence signal arises from endosomal release of DNA. Since our aim was investigating the intracellular dynamics of LFN/DNA complexes, we demonstrated that DNA was located inside endosomes (Fig. 2). We therefore detected the spot-like fluorescence signals from a region of interest inside the cell and used a specific tracking algorithm to identify and track complexes (for a detailed description of the tracking algorithm and its validation see the Methods section and Fig. S1 in the Supplementary Information). Briefly, trajectories are generated by extraction of the *x*- and *y*-coordinates of the complexes in the Cy-3 channel in all the acquired frames (Fig. 1A). For Lipofectamine/DNA complexes 122 trajectories are generated, corresponding to the movement of 29 complexes. Figure 1B shows a representative trajectory of a LNF/DNA complex followed for 5 min. Trajectory segmentation was performed by using a custom algorithm (for a detailed description of the segmentation algorithm and its validation see the Methods section and Fig. S2 in the Supplementary Information), which is able to identify trajectory domains characterized by Brownian diffusion (red) or flow motion (blue). The observed alternation of diffusion and flow motion domains reflects the stop-and-go motion characteristic for transport along microtubules^15^. Remarkably, after analysis of all trajectories, we are able to conclude that Brownian diffusion is the major route of intracellular trafficking of LFN/DNA complexes (64.3%) in CHO cells under physiological conditions (Fig. 1F). This is the first time that this type of motion is observed as the preferred mode of motion for LFN-based vectors trafficking towards the cell nucleus. As mentioned earlier, contrary views exist on this point. In more detail, Ondřej and colleagues showed that LFN/DNA complexes displayed directional motion toward the cell nucleus^7^. They observed strong binding of DNA-lipid complexes to the cytoskeleton and directional long-range motion of these lipoplexes along the microtubules. Disruption of this network led to the expected cessation of plasmid transport to the nucleus, a decreased mobility of plasmids, and accumulation of plasmid DNA in large aggregates at the cell periphery. It must be noted, however, that in those investigations both the DNA and the microtubule network were labeled and only complexes attached to the microtubule network were considered for tracking. This introduced a bias in the proposed conclusions on LFN mode of motion. By contrast, here we avoid any assumption about the complexes to be selected for the analysis. As a consequence of our measurement protocol, we are able to derive a complete scenario about how LFN/DNA complexes move within the cytoplasm. As control, DNA-loaded DD liposomes were tracked leading to 67 trajectories. The lipid/DNA ratio of DD/DNA complexes was selected according to a preliminary physical-chemical characterization (Fig. S3 in the Supplementary Information). In Fig. 1C an exemplary trajectory of a DD/DNA complex is displayed. Again, Brownian diffusion and active transport domains can be distinguished. Of note, however, in this case the proportion of DD/DNA complexes undergoing directed motion (71.9%) largely dominates as compared to LFN (25.7%). At this point we used nocodazole (NCZ) to elucidate the role of the cytoskeleton in the trafficking of complexes to the cell nucleus. NCZ belongs to a class of tubule-depolymerizing agents that bind specifically to tubulin and inhibit its polymerization^10^. As expected, NCZ treatment is able to disrupt the microtubule network in few minutes, as visualized by tubulin immunostaining (Fig. S4 in the Supplementary Information). While untreated CHO cells are elongated, with many detectable microtubules that run parallel to the long axis of the cell, NCZ-treated cells appear almost symmetrical (i.e. round-shaped) with only a few detectable microtubules. 32 LFN/DNA and 29 DD/DNA complexes were tracked in NCZ-treated CHO cells, leading to 47 and 51 trajectories, respectively. Noteworthy, LFN/DNA complexes were only slightly affected by NCZ treatment, i.e. they maintained a marked contribution from Brownian motion. By contrast, a major impact was observed for DD/DNA complexes: in this case the characteristic mode of motion clearly switches from flow motion to Brownian diffusion (Fig. 1G). This experiment supports the idea that flow motion takes place along microtubules. The presence of a residual percentage of flow motion in NCZ-treated CHO cells may reflect the active transport along actin filaments, as already reported by others^7^, rather than a contribution from the repolymerization of the microtubule network (Fig. S4 in the Supplementary Information). At this point, we are tempted to conclude that Brownian diffusion is an alternative, microtubule-independent, mechanism by which LFN/DNA complexes are able to move within the cytoplasm. Lastly, a global analysis of all the trajectories was performed. The mean square displacement (MSD) of fluorescent particles, a measure for the average distance a complex has traveled within a time lag Δt, was calculated as explained in the Supporting Information. By means of the MSD versus time plots (Fig. 1H) diffusion coefficient and velocity can be easily derived (see Table S1 in the Supplementary Information). The average velocity along the microtubules is v ≈ 0.45 μm/min for both formulations, while the diffusion coefficient of LFN (D_LFN_ ≈ 1.0 ± 0.2 × 10^−3^ μm^2^/s) was roughly 3-fold larger than that of DD (D_DD_ ≈ 3.7 ± 0.7 × 10^−4^ μm^2^/s). In keeping with previous findings^7^, the inhibition of tubulin polymerization reduces the average speed of actively transported LFN/DNA particles in the cytoplasm (v_LFN_ = 0.30 ± 0.03 μm/min). Their free diffusion also decreases by a factor >4 (D_LFN_ ≈ 2.3 ± 0.6 × 10^−4^ μm^2^/s). The same general trend was observed for DD (v_DD_ ≈ 0.39 ± 0.09 μm/min; D_DD_ ≈ 1.0 ± 0.1 × 10^−4^ μm^2^/s). The values of these dynamical parameters are similar to those measured for the dynamics of plasmids bound to actin filaments in untreated cells^7^. This further supports our suggestion that the residual percentage of active transport in NCZ-treated cells (Fig. 1G) is due to motion along the actin network.

### LFN-loaded vesicles are not destined to metabolic degradation

**Figure 3:**
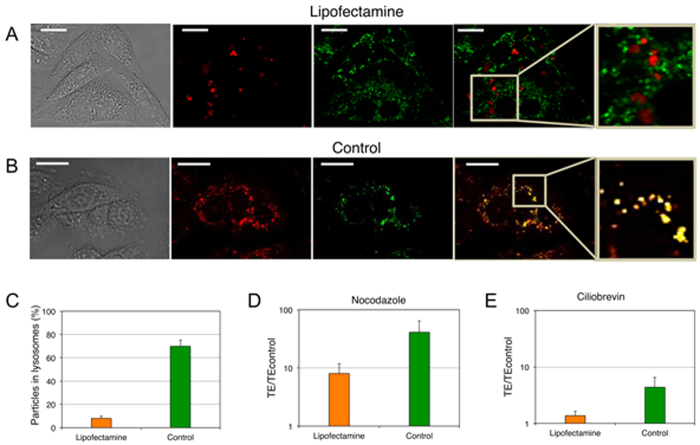
Colocalization of Lipofectamine (**A**) and DOTAP/DOPC liposomes (i.e. control) (**B**) loaded with Cy3-labeled DNA (red) with Lysosome marker (green). (**C**) Percentage of DNA-loaded particles in the lysosomes. Transfection efficiency (TE) of Lipofectamine/DNA and control complexes in nocodazole- (**D**) and Ciliobrevin (**D,E**) treated CHO cells with respect to TE measured in untreated cells (TE_control_).

To investigate the correlation between the mechanism of intracellular trafficking and the final fate of TRs, we labeled the CHO cells with Lysosensor, a primary lysosome marker. Two representative examples are displayed in Fig. 3. Figure 3A, in particular, shows that red fluorescence of LFN/DNA complexes does not colocalize with green fluorescence from Lysosensor. To obtain a quantitative estimation of the percentage of complexes trapped within lysosomes, an object-based colocalization analysis was used, as already explained elsewhere^16^. The colocalization analysis demonstrates that the vast majority of LFN/DNA complexes (≈90%) are able to avoid metabolic degradation into the acidic/digestive lysosomal compartments. By contrast, as illustrated in Fig. 3B, DD/DNA lipoplexes are clearly discernible as bright yellow dots, resulting from the colocalization of the red Cy3-labeled DNA and the green Lysosensor. Consequently, the quantitative colocalization analysis (Fig. 3C) showed that more than 70% of DD/DNA complexes are trapped within lysosomes. The interpretation is straightforward: since intact microtubules are required for the trafficking of endocytic vesicles towards lysosomes^17^, TRs that are preferentially transported along the microtubules are shuttled from endosomes to lysosomes (i.e. metabolic degradation) and are thus poorly efficient; on the contrary, LFN, by avoiding active transport along the cytoskeleton components, does evade lysosomal degradation, thus in turn increasing the probability of DNA release into the cytosol likely by the formation of multiple transient pores over time within the endosomal membrane^18^.

### Seeking the molecular basis of LFN intracellular transport

Now the question arises on whether inhibition of active transport may effectively result in a boost of TE, as expected from the evidences reported so far. To test this hypothesis, we evaluated the TE of LFN and DD on both untreated and NCZ-treated CHO cells (experimental details can be found in the Supporting Information). Figure 3D shows that the pre-treatment with 16 μM NCZ is able to increase the characteristic TE of LFN by a factor ≈8. Figure 1F shows that in one third of cases LFN is actively transported. So microtubules depolimeryzation could allow this fraction to diffuse, avoid degradation and boost TE. By contrast, in the case of DD/DNA complexes, transfection was increased approximately 40-fold.

We shall conclude that the efficiency of TRs which are able to exploit diffusion (i.e. LFN) does weakly rely on intact microtubules, contrary to the actively-transported ones (i.e. DD). TE is proportional to the number of plasmid copies delivered into the nucleus. Under NCZ treatment, LFN exhibits similar expression, i.e. a slightly higher number of gene copies must have reached the nucleus. In contrast, in case of DD, pretreatment of CHO cells with NCZ leads to a much higher number of DNA copies localized within the nucleus. This in turn indicates that, with respect to untreated cells, a higher number of plasmid copies was able to avoid trapping within lysosomes: we are prompted to correlate this result to the switch in the trafficking mechanism of the nanocarrier. At this point, it is legitimate to question about the molecular mechanisms regulating the interaction between TRs and microtubules. Most trafficking of particles along microtubules toward the nucleus uses the major minus-end-directed molecular motor dynein^15^. The mechanisms of particle-dynein complex formation are as yet largely unknown, but it is probable that they resemble those that regulate the interaction between dynein and multiprotein complexes that are transported to the nucleus along microtubules^19^,^20^. These interactions are controlled by specific protein such as importin-α, importin-β, nuclear localization signal (NLS)-containing proteins, spectrins etc. These proteins interact with membrane phospholipids through multiple sites^21^, with inositol lipids through the pleckstrin homology (PH) domain^22^, and with microtubule-based motors, either directly^23^, or through dynactin^24^. As such, we sought to determine how treatment of CHO cells with Ciliobrevin D, a pharmacological inhibitor of dynein^25^, could affect TE of TRs. Ciliobrevin D is an inhibitor of dynein ATPase activity, able to prevent the cycling activity of the motor protein. Figure 3E shows that pretreatment with Ciliobrevin D does not affect the characteristic TE of LFN, while it produces a detectable boost of TE for DD. These results indicate that the interaction of DOTAP and DOPC with dynein is more efficient than that occurring between dynein and the lipids that constitute LFN. This might be explained by differences in the binding mechanism, e.g. the higher affinity of DOTAP and DOPC for dynein with respect to lipids constituting LFN. It looks like LFN becomes ‘inert’ with respect to the binding machinery that is committed to active transport along microtubules. This ends up into random diffusion within the intracellular environment, a behavior reminiscent of that recently observed by some of us within the cytoplasm for small, inert, globular proteins^26^.

In conclusion, we demonstrate here that Brownian diffusion is an efficient route for LFN/DNA complexes to avoid metabolic degradation, thus leading to optimal transfection. By contrast, active transport along microtubules results in DNA degradation and subsequent poor transfection. In this view, intracellular trafficking, endosomal escape and lysosomal degradation can be viewed as interdependent phenomena, in such a way that they appear as a single barrier on the route for efficient transfection. Our conclusions were possible by the combination of visualization and analysis techniques we have used which make no assumption about the mechanism of transport of LFN.

## Methods

### LFN/DNA complexes

LFN/DNA complexes were prepared according to the manufacturer’s instructions. Briefly, complexes were prepared in Optimem (Invitrogen, Carlsbad, CA) by mixing for each well of 6-well plates 2.5 μg of Cy-3 plasmid (Invitrogen, Carlsbad, CA) with 5 μl of Lipofectamine 2000 (LFN 2000) transfection reagent (Life Technologies, Carlsbad, CA). Before complexation, DNA and LFN were diluted with 197.5 μl and 195 μl of Optimem. Complexes were left for 20 min at room temperature (RT) before adding them to the cells.

### DOTAP/DOPC/DNA complexes

The cationic lipid 1,2-dioleoyl-3-trimethylammonium-propane (DOTAP) and the zwitterionic helper lipids dioleoylphosphocholine (DOPC) were purchased from Avanti Polar Lipids Inc. (AL, USA) and used without further purification. DOTAP/DOPC (DD) cationic liposomes were routinely prepared^27^,^28^,^29^,^30^,^31^. In brief, a lipid mixture, at molar fraction of neutral lipid ϕ = neutral/(neutral + cationic) (mol/mol) = 0.5, was dissolved in chloroform and then left to evaporate under vacuum for at least 24 h. The obtained lipid film was hydrated with nanopure water (1 mg/ml). Sonication to clarity of the obtained dispersion was performed to obtain unilamellar cationic liposomes. To identify the most appropriate cationic lipid/DNA charge ratio (ρ) for transfection, size and zeta-potential of DD/DNA complexes were measured at RT by a Malvern NanoZetaSizer spectrometer equipped with a 5 mW HeNe laser (wavelength λ = 632.8 nm) and a digital logarithmic correlator. The normalized intensity autocorrelation functions were analyzed by using the CONTIN method, which analyzes the autocorrelation function through an inverse Laplace transform and provides the distribution of the diffusion coefficient D of the particles^32^. The diffusion coefficient is converted into an effective hydrodynamic radius R_H_ by using the Stokes-Einstein equation R_H_ = K_B_T/(6πηD), where K_B_T is the thermal energy and η the solvent viscosity. Electrophoretic measurements allowed to measure the mobility u, which was converted into the Zeta-potential using the Smoluchowski relation zeta-potential = uη/ε, where η and ε are the viscosity and the permittivity of the solvent phase, respectively. For size and zeta-potential measurements samples were prepared at the thirteen cationic lipid/DNA charge ratios (mol/mol), ρ. reported in Table S1. In Fig. S3 the hydrodynamic diameter (D) and the zeta-potential of DD/DNA lipoplexes are plotted against ρ. For 0.5 < ρ < 4, complexes were found to be negatively charged and both their size and zeta-potential increased with increasing ρ. At ρ ~ 5 complexes were neutrally charged (zeta-potential ~0 mV) with the largest size (D ~ 2 μm). Further increase of the lipid content (ρ > 5) induced charge inversion and re-entrant condensation of complexes^33^. On the basis of size and zeta-potential results, all the confocal microscopy experiments were performed at ρ = 6. This charge ratio allows obtaining positively charged complexes with the minimum colloidal dimensions.

### Cell culture

CHO-K1 were purchased from American Type Culture Collection (CCL-61 ATCC) and were grown in Ham’s F12K medium supplemented with 10% of fetal bovine serum at 37 °C and in 5% CO_2_. The day before transfection, cells were seeded in 6-well plates using medium without antibiotics. Cells were incubated until they were 75–80% confluent, which generally took 18–24 h. LFN/DNA and DD/DNA complexes were prepared in Optimem (Invitrogen) as explained below. Complexes were left for 20 min at room temperature before adding them to the cells. Cells were incubated at 37 °C for an additional 4 h to permit complex internalization. Finally, cells were extensively washed three-times with phosphate buffered saline (PBS) before DMEM medium supplemented with 10% fetal bovine serum at 37 °C was added. To depolymerize microtubules, Nocodazole (NCZ) was used. NCZ was purchased from Sigma-Aldrich. To investigate the dynamics of complexes in NCZ-treated cells, the transfection procedure was slightly different. Before lipoplex administration, cells were first incubated for 15 min with a 16-μM NCZ medium and then washed three-times with 2 ml of PBS. According to preliminary transfection experiments (not reported) such drug concentration was the highest possible concentration that did not significantly reduce cell viability.

### Single Particle Tracking analysis

Confocal microscopy experiments were performed with the Olympus Fluoview 1000 (Olympus, Melville, NY) confocal microscope interfaced with a 405 nm diode laser, a 488 nm argon laser, and 543 nm helium−neon laser. The cytoplasmatic trafficking of LFN/DNA and DD/DNA complexes has been explored both in un-treated and NCZ-treated CHO cells by Single Particle Tracking (SPT). The fluorescent signal of Cy3-labeled nucleic acids allowed us to follow the dynamics of the nanocarriers for a period T (2 min ≤ T ≤ 5 min) at a sampling rate τ^−1^ = 1 frame/s. We preliminary checked that global drifts of the cells did not affect the time evolution of the fluorescence spots. If a global motion of cells was detected, then that acquisition was discarded. Finally, the fluorescence images have been detected from a region of interest at different times (t_i + 1_ = t_i_ + τ), collected in sequences and the bidimensional trajectory (x_j_(t), y_j_(t)) of the j-th particle has been evaluated through a specific tracking algorithm. For the particle identification and the trajectory evaluation, we adopted TrackMate, a proper package in an open source image processing software (i.e. Fiji), which provides the tools to perform single particle tracking (SPT). The fluorescent spot-like structures are represented by (x, y, t) coordinates arrays, size parameters and orientation. The adopted detector for the particle identification uses the difference of Gaussians (DoG) approach and the tracking algorithm is based on the Linear Assignment Problem (LAP), which allows dealing with gap-closing events. The settings have been optimized to prevent splitting and merging events. By merging the bright field and the fluorescence channels, we distinguished the spots of interest and excluded particles moving on the cell membrane or outside it, since they are not involved in the analysis of the cytoplasmic trafficking. Furthermore, undesired tracks have been discarded using proper filters, acting on starting positions, tracking qualities and total track lengths. Finally, for each particle, the time evolution of the positions has been exported and processed through custom Matlab codes. The track analysis over the entire acquisition time was firstly carried out by evaluating the Mean Square Displacement (MSD). It is defined as


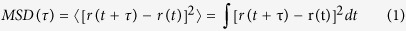


and it is related to the probability P(r’|r, τ) that a particle originally at r(t) will be at r’ after a time lag τ. If Brownian diffusion and flow motion transition probabilities are assumed, the bidimensional MSD can be expressed respectively in the following forms:


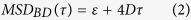



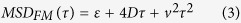


where D represents the diffusion coefficient, v is the active transport speed and ε is a positive offset due to the localization uncertainty. We therefore computed the MSD(τ) from the acquired tracks, fitted the data through the functional relationships 2–3, distinguished the kind of motion and evaluated the dynamic parameters (i.e. flow speeds and diffusion coefficients). Since the numerical computation of the MSD intrinsically suffers the low statistical significance at long τ, we restricted the analysis to τ values lower than 10% of the total track’s time, in agreement with common SPT criterions^34^. Furthermore, we measured the asphericity parameter α on the ensembles and on single tracks, in order to consolidate the MSD analysis and characterize diffusive or flow motion behaviors whether they were not easily distinguishable from the MSD data fitting. Moreover, we measured the asphericity parameter α to consolidate the MSD analysis and characterize the tracks whose diffusive or flow motion behaviors were not easily distinguishable from the MSD data fitting. α is related to the global “shape” of the track (i.e. α = 0 for strongly confined motion, α = 1 for totally directed motion) and it has been evaluated from the eigenvalues of the gyration tensor T_ij_.


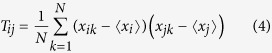


where N is the number of acquired frames, x_ik_ is the i-th component of the position vector of the k-th point and the brackets identify the average over the trajectory. For d-dimensional trajectories


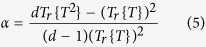


The theoretical average asphericity for bidimensional Brownian processes is α_Br_ = 0.57^35^. If only one track is considered, the single particle asphericity α_sp_ can be measured from the corresponding tensor, just like α for an ensemble made of a single unit. Fig. S1 shows the distributions of α_sp_, measured from numeric simulations of Brownian Diffusion and Brownian Diffusion + Flow Motion. We generated ensembles of 10^5^ tracks, whose dynamic parameters are those experimentally determined from the MSD of the systems in not treated cells. Our results of the global analysis can be summarized as follows: (i) The dynamics of nanoparticles in nocodazole-treated cells is generally driven by Brownian Diffusion (linear fit of the MSD and low α values); (ii) flow motion represents a relevant contribution to the cytoplasmatic trafficking of the control nanoparticles in not treated cells (parabolic trend of the MSD and high α-value: α = 0.90); (iii) the MSD of Lipofectamine includes a quadratic term (Flow Motion), but the asphericity is very close to the theoretical diffusive value. A deeper insight on these behaviors can be achieved by local analyses of the tracks, which have been carried out by a proper segmentation algorithm. It allowed us to overcome the incoherence of the Lipofectamine results and improved the intracellular trafficking characterization. It is developed from the gyration quantification, which is recognized as a very accurate method for motion classification. Indeed, the aim is to distinguish diffusive and flow motion domains inside a single trajectory. The adopted criterion is based on successive segmentations and comparisons of local parameters (average speeds and asphericities) with threshold values, which have been set according to experimental and theoretical arguments. Our adopted approach is based on (i) partitioning the trajectories in m-length segments (m = 3) (Fig. S2A,B), (ii) averaging the speed components v_x_, v_y_ over the segments (Fig. S2C), (iii) evaluating the ensemble speed distribution (Fig. S2D,E) to determine the v-threshold values and obtain a partition in sequences of consecutive segments, then (iv) calculating the local asphericity over these sequences (Fig. S2F). The ensemble speed distributions have been evaluated over all the trajectories of a given formulation. We focused on the maximum values of |v_x_| and |v_y_| and set the thresholds v_x_^(thr)^, v_y_^(thr)^ as the 70% of the distribution averages. The local asphericities have been computed over the sequences of consecutive segments whose speed mean values belong to the threshold ranges and over consecutive segments whose speed mean values are outside the threshold ranges. Finally, a sequence of two or more consecutive segments has been classified as a flow motion domain if its local asphericity α_L_ overcome a fixed threshold value, α_thr,_ which is related to the probability 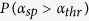 that a 2-dimensional Brownian process yields 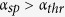. This can be computed by integrating the single particle asphericity distribution 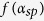, which is obtained from numerical simulations.


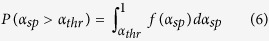


We set α_thr_ = 0.8, numerically computed the integral in Eq. 6 and found out that for 2-dimensional Brownian processes, the probability to have single particle asphericities higher than 0.8 is in the range 3–8% (depending on the track length). In conclusion, the adopted techniques allowed us to globally and locally analyze the particle tracks, measure the dynamic parameters (Table S2) and evaluate the relative populations of particles undergoing diffusion and flow motion, for each of the investigated systems.

### Colocalization analysis

Glass bottom Petri dishes containing transfected cells were mounted in a temperature-controlled chamber at 37 °C and 5% CO_2_ and viewed with a 60 × 1.25 numerical aperture (NA) water immersion objective. The following collection ranges were adopted: 555–655 nm (Cy3), and 460–530 (Lysosensor). Images were collected in sequential mode to eliminate emission cross talk between the various dyes.

### Transfection efficiency experiments

Cell lines were cultured in Dulbecco’s modified Eagle’s medium (DMEM) with Glutamax-1 (Invitrogen, Carlsbad, CA, USA) supplemented with 1% penicillin–streptomycin (Invitrogen) and 10% fetal bovine serum (Invitrogen) at 37 °C and 5% CO_2_ atmosphere, splitting the cells every 2–4 days to maintain monolayer coverage. The day before transfection, cells were seeded in 24-well plates (200,000 cells per well) using medium without antibiotics. For TE experiments, Lipofectamine/DNA and DOTAP/DOPC/DNA complexes were prepared in Optimem (Invitrogen) by mixing for each well of 24-well plates 0.5 μg of plasmid with 2 μl of Lipofectamine 2000 transfection reagent or (5) μl of DOTAP/DOPC sonicated lipid dispersions (1 mg/ml). Before complexation, DNA was diluted with (50) μl of Optimem, Lipofectamine with (48) μl of Optimem and DOTAP/DOPC liposomes with (45) μl of Optimem. Complexes were left for 20 min at room temperature before adding them to the cells. On the day of transfection, the growth medium was replaced with 400 μl of Optimem and the cells were incubated for 30 min at 37 °C, before adding complexes (100 μl/well). Cells were incubated at 37 °C for an additional 3 h to permit transient transfection. After (24) h, cells were analyzed for luciferase expression using Luciferase Assay System from Promega. Briefly, cells were washed in PBS and harvested in 200 μl 1× reporter lysis buffer (Promega). Of the cell suspension, 20 μl was diluted in 100 μl luciferase reaction buffer (Promega) and the luminescence was measured 10 s using a Berthold AutoLumatluminometer LB-953 (Berthold, Bad Wildbad, Germany).

## Additional Information

**How to cite this article**: Cardarelli, F. *et al*. The intracellular trafficking mechanism of Lipofectamine-based transfection reagents and its implication for gene delivery. *Sci. Rep.* **6**, 25879; doi: 10.1038/srep25879 (2016).

## Supplementary Material

Supplementary Information
