# Supplementary material for: The intracellular trafficking mechanism of Lipofectamine-based transfection reagents and its implication for gene delivery
Source: Sci Rep. 2016 May 11;6:25879. doi: 10.1038/srep25879 (PMC4863168; doi:10.1038/srep25879)
Supplement: Supplementary Information [file srep25879-s1.pdf]

Supplementary Information

The intracellular trafficking mechanism of Lipofectamine-based transfection reagents and its implication for gene delivery

Francesco Cardarelli,[a#] Luca Digiacomo,[b,c#] Cristina Marchini,[b] Augusto Amici,[b] Fabrizio Salomone,[a] Giuseppe Fiume,[a] Alessandro Rossetta,[d] Enrico Gratton,[d] Daniela Pozzi,[c] Giulio Caracciolo[c*]

[a]*Center for Nanotechnology Innovation @NEST, Istituto Italiano di Tecnologia, Piazza San Silvestro 12, 56127 Pisa, Italy*

[b]*School of Bioscience and Veterinary Medicine, University of Camerino, Via Gentile III da Varano, 62032 Camerino (MC), Italy*

[c]*Department of Molecular Medicine, "Sapienza" University of Rome, Viale Regina Elena 291, 00161, Rome, Italy*

[d]*Laboratory for Fluorescence Dynamics, Department of Biomedical Engineering, University of California, Irvine, 3120 Natural Sciences 2, Irvine, CA 92697-2715, USA*

Table S1. Cationic lipid/DNA charge ratios (mol/mol), ρ, used to prepare DOTAP/DOPC/DNA complexes for size and zeta-potential experiments.

	ρ
**1**	1.1
**2**	2.2
**3**	3.2
**4**	3.8
**5**	4.3
**6**	4.8
**7**	5.0
**8**	5.1
**9**	5.2
**10**	5.4
**11**	5.7
**12**	5.9
**13**	6.5

Table S2. Dynamic parameters of the investigated delivery systems, measured through SPT analysis (Mean ± SD values are displayed).

	**Not treated cells**			**Nocodazole-treated cells**		
	**Diffusion**	**Flow motion**		**Diffusion**	**Flow motion**	
	D (nm^2/s)	v (nm/s)	D (nm^2/s)	D (nm^2/s)	v (nm/s)	D (nm^2/s)
**Lipofectamine**	984±179	7.5±1.3	549±155	226±60	4.9±0.5	356±101
**Control**	370±68	7.4±1.1	255±66	103±13	6.6±1.5	252±103

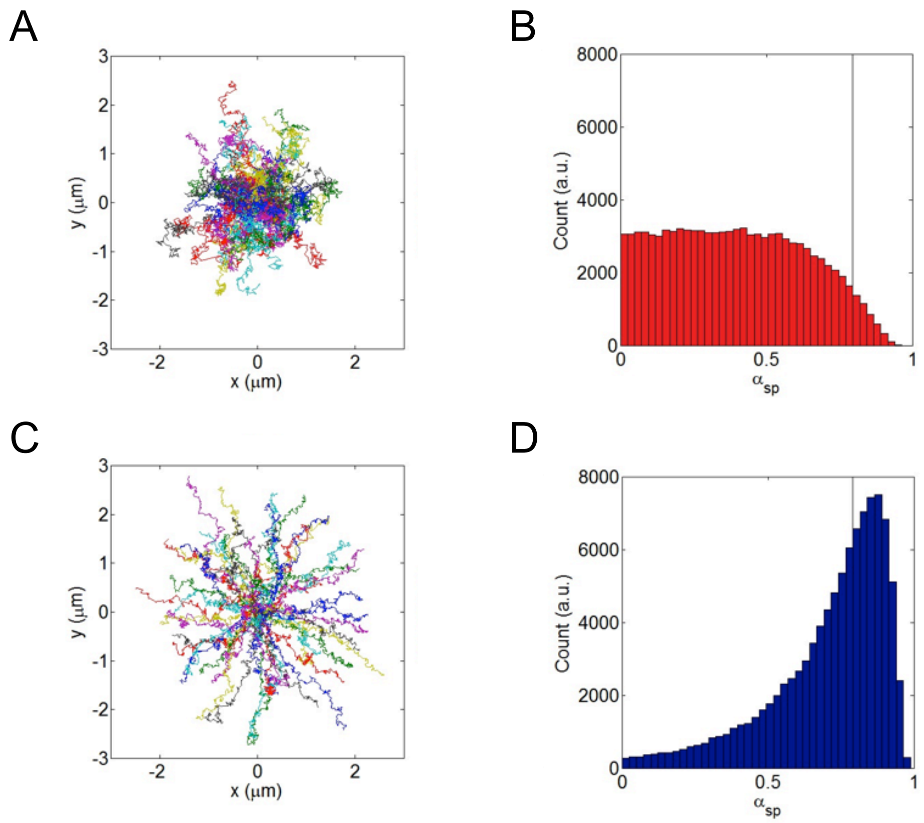

Fig. S1. Validation of categorization algorithm. (A) Representative tracks obtained from simulations of Brownian Diffusion and (B) relative single particle asphericity distribution. (C) Representative tracks obtained from simulations of Brownian Diffusion + Flow Motion and (D) relative single particle asphericity distribution. Each ensemble is made of 100.000 trajectories each of them made of 300 frames. In simulations diffusion coefficients and flow speed have been set according to the MSD fits of LFN and DD complexes in not treated cells. Solid lines in panels B and D indicate the asphericity threshold values α_{thr}. The probability that a Brownian process yields $\alpha_{sp} > \alpha_{thr}$ is 3-8%. This can be assumed as final validation of the goodness of the categorization algorithm.

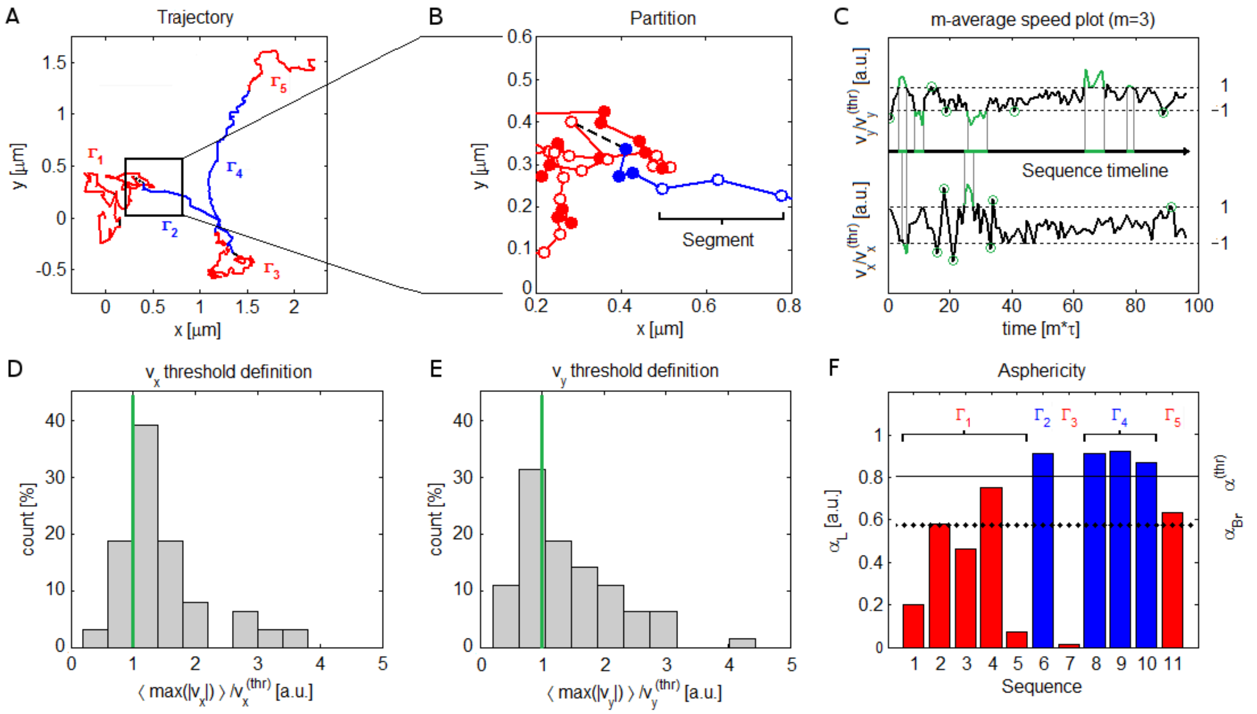

Fig. S2. Segmentation algorithm. Representative trajectory (A) and scheme of partition in m-length segments with m=3 (circles and dots in Panel B). The segments have been collected in sequences, by comparing the m-average speed (C) with the threshold values determined by the experimental ensemble distribution (E, F). Finally, the local asphericity over the sequences defines the diffusion (red) and flow motion (blue) domains.

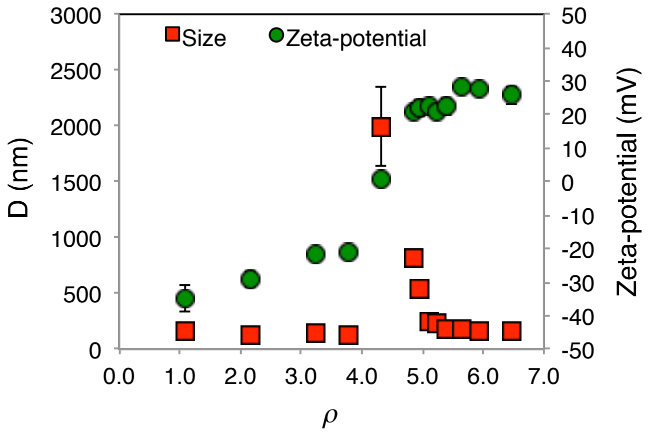

Fig. S3. Phenomenology of aggregation of DNA and DOTAP/DOPC cationic liposomes. Hydrodynamic diameter, D, and zeta-potential of DOTAP/DOPC/DNA complexes as a function of the cationic lipid/DNA ratio.

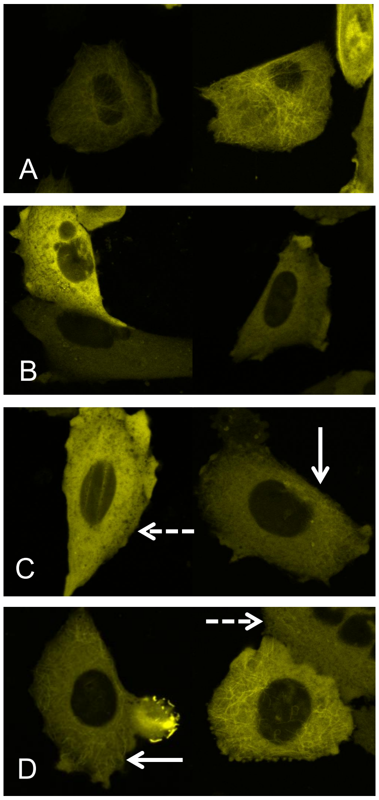

Fig. S4. **Tubulin cytoskeleton depolymerization and recovery.** Tubulin cytoskeleton staining of Chinese Hamster Ovary cells by Tubulin-Yellow Fluorescent Protein (panel A). Recovery of the microtubule network after Nocodazole-treatment: t=0 (panel B); t=4h (panel C); t=8h (panel D). Solid lines indicate newly formed microtubules, while dotted lines indicate regions of the plasma membrane where only poorly formed, if any, microtubules can be seen.
